# Immune cell dynamics, cytokines, and extracellular vesicles in systemic inflammatory response syndrome (SIRS): a multiparametric analysis

**DOI:** 10.1186/s12967-025-07327-z

**Published:** 2025-11-21

**Authors:** Gerardo J. Martí-Chillón, Sandra Muntión, Juan Flores-Montero, Javier González-Robledo, Luís Corchete, Julio Pozo, Lika Osugui, Silvia Preciado, Martín Gómez-Redondo, Juan F. Blanco, Almudena Navarro-Bailón, Víctor Sagredo, Julia Almeida, Alberto Orfao, Fermín Sánchez-Guijo

**Affiliations:** 1 Cell Therapy Area, University Hospital of Salamanca, Salamanca, Spain; 2 Hematology Department, University Hospital of Salamanca, Salamanca, Spain; 3 Faculty of Medicine, University of Salamanca (USAL), Salamanca, Spain; 4 Biomedical Research Foundation of Salamanca (FIBSAL), Salamanca, Spain; 5 Cytometry Service (NUCLEUS), University of Salamanca, Salamanca, Spain; 6 Network Center for Regenerative Medicine and Cellular Therapy of Castilla y León, Salamanca, Spain; 7 Intensive Medicine Department, University Hospital of Salamanca, Salamanca, Spain; 8 Orthopedics Department, University Hospital of Salamanca, Salamanca, Spain; 9 Translational and Clinical Research Program, Cancer Research Center (CIC, CSIC – University of Salamanca), Salamanca, Spain; 10 Biomedical Research Networking Centre Consortium of Oncology (CIBERONC), Instituto de Salud Carlos III, Madrid, Spain

**Keywords:** SIRS, Polytrauma, Sepsis, Biomarkers, Inflammation, Flow cytometry, Cytokine storm, Secretome, EVs, microRNA

## Abstract

**Background:**

The systemic inflammatory response syndrome (SIRS) is a complex and multifactorial life-threatening reaction triggered by trauma or infection (sepsis). Since the dynamics of immune cells, cytokines and novel markers such as circulating extracellular vesicles (EVs) remain incompletely elucidated, we aimed to provide a comprehensive multiparametric characterization of this severe response, to identify potential prognostic and therapeutic targets.

**Methods:**

We conducted a multiparametric and sequential quantification of up to 25 immune cell circulating populations using next-generation flow cytometry, assessed plasma cytokine levels with Luminex technology, and the characterized and quantified EVs and their microRNA content. Peripheral blood samples were taken at 0, 24 and 72 h after SIRS onset from 25 patients with polytrauma-induced SIRS (PT group) and 25 developed SIRS from other origins, such as pulmonary or urinary infections (NP-denoting non-polytrauma group). Samples from 21 healthy donors (HD) were analysed as a control group. Statistical analyses were performed to compare experimental groups (PT, NP, HD) and to evaluate correlations between experimental findings and patients’ outcomes for potential prognostic markers discovery.

**Results:**

SIRS patients exhibited increased leukocyte counts driven by neutrophil mobilization combined with a reduction of dendritic cells and lymphocytes early after the SIRS. The PT group showed an increase in classical monocytes, myeloid-derived suppressor cells (MDSCs), and mesenchymal stromal cells compared to HD. These dynamics occurred alongside substantial chemokine release, including IL-8, G-CSF, CCL2 in both SIRS groups, MIF in PT and CCL4 in NP. Both SIRS groups experienced a pronounced cytokine storm with simultaneous release of both pro- and anti-inflammatory cytokines, and increased of plasma EV levels. Our findings suggest that elevated IL-8 concentrations and increased counts of circulating monocytes and MDSCs are associated with worse prognosis and may serve as valuable prognostic indicators in SIRS patients.

**Conclusions:**

Our study demonstrates the dynamic kinetics of multiple cell subsets, cytokines and EVs during SIRS, highlighting differences between SIRS initiated by polytrauma versus non-polytrauma events and identifying correlations between biological findings and patient outcomes.

**Supplementary Information:**

The online version contains supplementary material available at 10.1186/s12967-025-07327-z.

## Background

Polytrauma and severe infections are among the most common critical healthcare issues worldwide [1], with incidence and mortality remaining high despite advances in management and treatment [2]. Many patients admitted to the intensive care unit (ICU) deteriorate due to both the progressive damage caused by the initial insult and the subsequent disfunction arising from the secondary inflammatory response. In critical cases, local inflammation can escalate into a systemic inflammatory response syndrome (SIRS, or sepsis if it derives from an infection) after the insult [3]. This condition can progress into a shock and multiple organ dysfunction syndrome (MODS), critically compromising the patient’s survival.

The inflammatory response is initiated by the release of damage-associated molecular patterns (DAMPs, or those related to pathogen molecules – PAMPs) from affected tissues. This signalling triggers immune activation, leading the secretion of multiple cytokines and extracellular vesicles (EVs) that induce nonspecific cell recruitment [4–6]. Some studies have described the distribution of major immune populations in different clinical conditions (sepsis, burns, major surgeries) [7–9]. However, the dynamics and the multiparametric assessment of key actors of this complex response such as inflammatory monocytes, myeloid-derived suppressor cells (MDSCs) or mesenchymal stromal/stem cells (MSCs) have not been reported in depth. In addition, the analysis of the non-cellular key players of SIRS, such as the secretome components (cytokines, extracellular vesicles) also warrants attention. Notably, this work provides a multiparametric, dynamic and sequential assessment of key actors of this inflammatory response, not described to date. In this sense, a dynamic and multi-omics-based analysis of this complex inflammatory reaction would be crucial for elucidating new prognostic markers and therapeutic targets. Furthermore, we have also considered that the potential differences in SIRS cases initiated after polytrauma compared to those of non-polytrauma origin may provide additional insights with potential clinical relevance.

## Methods

In the current work, we have identified and quantified more than 25 circulating cell subsets and major secretome components (cytokines, chemokines and EVs) at different time points from 50 ICU patients according to the SIRS origin (polytrauma [PT] versus non-polytrauma [NP]), and from healthy donors (HD). To achieve this, advanced flow cytometry, Luminex multiplex assay and EV analytical platforms were used to dissect the dynamics of immune cells and secretome mediators.

### SIRS patients and control samples

The study was conducted in accordance with the Declaration of Helsinki guidelines and local ethics IRB (CEIm Area de Salud de Salamanca) approved the study (ref. code 201912400) before patient enrollment. Prior to peripheral blood (PB) collection, participants (or their relatives in those who were incapacitated) signed a written informed consent form. We included 50 patients fulfilling the following criteria: ≥ 2 SIRS criteria, >18 years old, lack of immunosuppressive therapy prior to ICU admission, absence of concurrent viral infections (SARS-CoV-2, HIV) or hematological malignancies. Half of the patients (*n* = 25) presented SIRS after a polytrauma (traffic accidents or falls injuries) and the rest of the patients (*n* = 25) derived from other origins (urinary infections, pneumonia, pancreatitis, mainly). Relevant clinical information about patients is summarized in the Table S1 (see Additional File 1). PB samples (5–10 mL, in ethylenediaminetetraacetic acid [EDTA] and heparin tubes) from the 50 patients admitted to the ICU were obtained at 0, 24, and 72 h after SIRS onset, and all samples were processed during the first 8 h after collection. PB samples from HD were used as the control group (*n* = 21). Following informed consent, additional blood was drawn during a routine blood test for research purposes.

### Sample processing and flow cytometry analysis

The cellular component of each EDTA-anticoagulated PB sample was separated from the plasma after centrifugation at 800 g for 10 min. Ammonium chloride-based erythrocyte lysis was performed following EuroFlow standard operating protocols (www.euroflow.org) [10]. Briefly, fresh PB after patient enrolment was treated with 1X BulkLysis solution (BD/Cytognos SL, Salamanca Spain; Ref. CYT-BL) for 15 min at room temperature and centrifuged for erythrocyte debris removal. Around 10-20 × 10^6^ nucleated cells were stained using monoclonal antibodies of the 14-color MoDC myeloid panel [11] with Brilliant Stain Buffer (Becton Dickinson Biosciences, BD; Ref. 563794). For studying the lymphoid populations, 5-10 × 10^6^ cells were labelled with the 8-color EuroFlow LST combination (OneFlow Lymphoid Screening Tube, BD ref. 658619) [12]. The specifications of the conjugated antibodies and fluorochromes used for each panel are detailed in the Table S2a (Additional File 1) [12, 13].

For circulating MSCs identification, a similar protocol was followed. Approximately 20-30 × 10^6^ cells were lysed with BulkLysis solution. Cells were quantified and stained with 1 µl of the cell viability marker Fixable Viability Stain 700 (BD ref. 564997) and then incubated with the antibodies specified in the Table S2a (Additional File 1).

Cells were acquired on a FACSymphony A5 flow cytometer (BD) for both MoDC and MSCs tubes and a FACSLyric flow cytometer (BD) for the LST tube. FACSDiva and FACSuite software were used for data acquisition, respectively. Sample acquisition was performed after the appropriate cytometer setup, calibration and specific compensation were performed following EuroFlow recommendations [10]. Infinicyt™ v.2.0 software (BD/Cytognos SL) was used for the analysis of the cytometry data. For cell identification and enumeration, previously defined gating strategies were followed [13, 14]. The Table S2b (Additional File 1) informs about the markers used for identifying each population. Quantification of leukocyte populations per millilitre of blood was calculated by extrapolating values from the total white blood cell counts in the automated hemocytometer. A cellular population was defined as a cluster of ≥ 10 cells meeting the specific immunophenotype for every 10 × 10^6^ acquired events. Furthermore, the expression (median fluorescence intensity – MFI) of HLA-DR and CD62L (CD - cluster of differentiation) was studied in the MoDC tube.

The gating strategy used for the identification of circulating MSCs after eliminating dead cells based on their positivity to Viability Stain 700 labeling and excluding doublets using the FSC-A/FSC-H diagram. Subsequently, the population of circulating hematopoietic progenitor cells (CD34^pos^, CD45^dim^) was identified, used as a reference for subsequent analyses. Next, the population meeting the MSC immunophenotypic profile criteria proposed by the International Society for Cell and Gene Therapy (CD34^neg^, CD45^neg^, CD73^pos^, CD90^pos^, CD105^pos^, CD13^pos^, and HLA-DR^neg^) was selected [15].

### Plasma cytokine quantification

To determine the concentration of cytokines the Luminex platform (R&D Systems, Minneapolis, MN) was used. For this purpose, we used the plasma after the previously mentioned centrifugation step (800 g for 10 min), after which the supernatant was frozen at -80 °C for subsequent analyses. The supernatant was then diluted 1:2 and incubated subsequently with specific reagents following the manufacturer’s instructions. Alarmins (S100A8, sRAGE), pro-inflammatory cytokines (IFNγ, TNFα, IL-1β, IL-2, IL-12, IL-18), anti-inflammatory/regulatory cytokines (IL-4, IL-10, IL-6), chemokines (IL-8, CCL2/MCP-1, CCL3/MIP-1a, CCL4/MIP-1b, CXCL1/GROα, MIF and G-CSF) and other factors (GM-CSF, VEGF, FASL), were measured in a Luminex 100/200™ analyser (Bio-Techne, R&D Systems).

### EVs characterization and study of EV-microRNA content

EVs were obtained following the collecting and pre-processing recommendations proposed by the International Society for Extracellular Vesicles MISEV2018 and MISEV2023 for EVs from biofluids [16, 17]. Heparin tubes were used for the study of the EV morphology by using Transmission Electron Microscopy (TEM), size determination and particle quantification by Nanoparticle Tracking Analysis (NTA), and protein expression by Western Blot (WB). Plasma samples from EDTA tubes were randomly selected within each group for the study of the microRNA (miRNA) content. For all these approaches, the isolation of EVs was performed following conventional ultracentrifugation, as previously described [18] (Table S3, Additional File 1). To study the miRNA content, EVs were isolated from a minimum of 4 mL of plasma from 6 HD and 21 SIRS patients (11 PT and 10 NP samples from timepoint 0 h). Additional information on the protocols followed for EV characterization and EV-miRNAs content analysis are compiled in the Table S3 (Additional File 1).

### Statistical analysis

Statistical analyses were performed using SPSS version 23 and R Studio 2025.05.1 + 513, and GraphPad Prism v.6 for graphics. The median was used as the central tendency value for a set of data (group) and the interquartile range as a measure of statistical dispersion. The non-parametric Mann-Whitney U test was used for intergroup comparisons at each time. The Wilcoxon test was used for studies of kinetics or changes in two times of dependent samples. Qualitative data were compared using Fisher’s test for independent samples. Spearman correlation analysis was used to measure the relationship grade among the studied variables. The determination of cut-off points for subgrouping studies according to a variable was determined by Receiver Operating Characteristic (ROC) curves (considering sensitivity and specificity parameters). In addition, the Kaplan-Meier model was used to estimate patient survival and analysed with a log-rank test based on chi-square (χ²) statistic. Potential confounders were analyzed using both the APACHE II and SOFA scores to ensure appropriate adjustment in the analysis. Results with p-value ≤ 0.05 were considered statistically significant following: *p ≤ 0.05, **p ≤ 0.01, ***p ≤ 0.001.

## Results

### Distribution and kinetics of total leukocytes and granulocytes

**Fig. 1:**
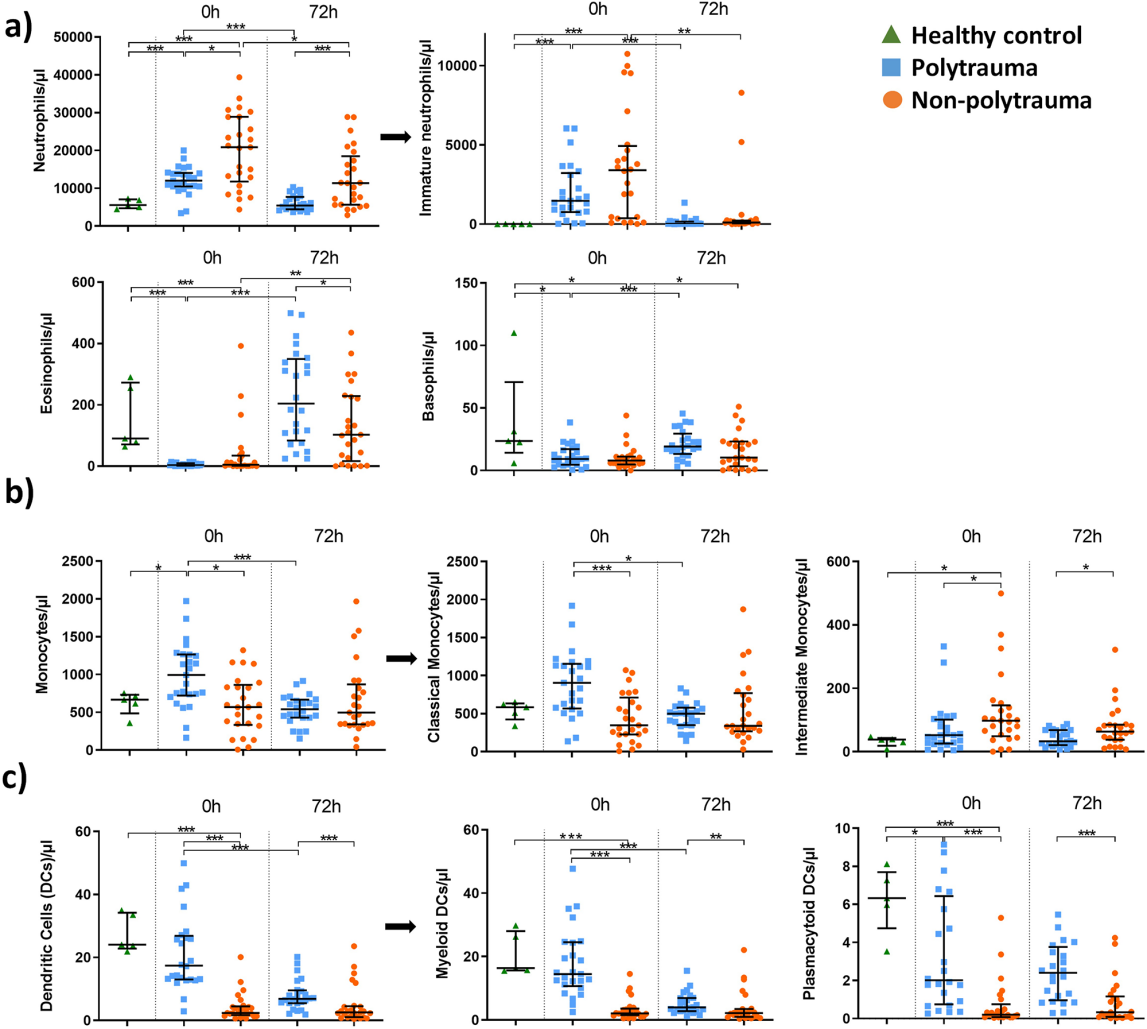
Kinetics of major myeloid circulating population in SIRS patients. (**a**) Results of granulocyte counts, in which neutrophils represented up to 85–95% of total leukocytes at 0 h in most SIRS patients, (**b**) total monocytes and classical and intermediate subsets, (**c**) DCs (dendritic cells) and major subsets (myeloid and plasmacytoid)

Regarding cytometric results, total leukocytes increased after SIRS onset in both SIRS groups, being almost PT 2-fold and NP 3-fold higher than HD. PT group returned to normal values after 72 h, meanwhile in NP group leukocyte counts remained increased. This increase was directly determined by a massive mobilization of neutrophils (both mature and banded subsets) (Fig. 1a). In contrast to this increase, other granulocyte populations such as eosinophils and basophils count decreased.

### Monocytes and dendritic cells kinetics in peripheral blood

Regarding other myeloid populations, total monocytes remained balanced during SIRS but increased acutely in the PT group. This increase was due to the mobilization of the classical monocyte’s subset (cMo, CD14^pos^CD16^neg^) (Fig. 1b). NP patients showed an increase in the intermediate or inflammatory monocytes (iMo, CD14^pos^CD16^pos^) that remained high at 72 h (Fig. 1b). Both SIRS groups showed a decrease in the non-classical monocytes (ncMo, CD14^neg^CD16^pos^), but it was only statistically significant between PT and HD at 0 h (data not shown). Subsets of ncMo defined by CD36/Slan expression showed a similar decrease.

Dendritic cells (DCs) decreased in both groups and more acutely in NP patients (Fig. 1c). This kinetic was followed by all the major DCs subpopulations. Myeloid origin DCs (mDCs) and plasmacytoid (pDCs) remained low after 72 h. In-depth analysis of mDCs subsets according to the expression of CD141 or CD5/CD14 showed a reduction in these subsets in both SIRS groups compared to HD (data not shown).

HLA-DR is a key marker in the activation of the adaptative immune cells. The expression of HLA-DR in antigen-presenting cells (APC, monocytes and DCs) decrease during SIRS (Figure S1a, Additional File 1), especially in the monocyte population in both pathological groups.

### Kinetics of major lymphoid populations

**Fig. 2:**
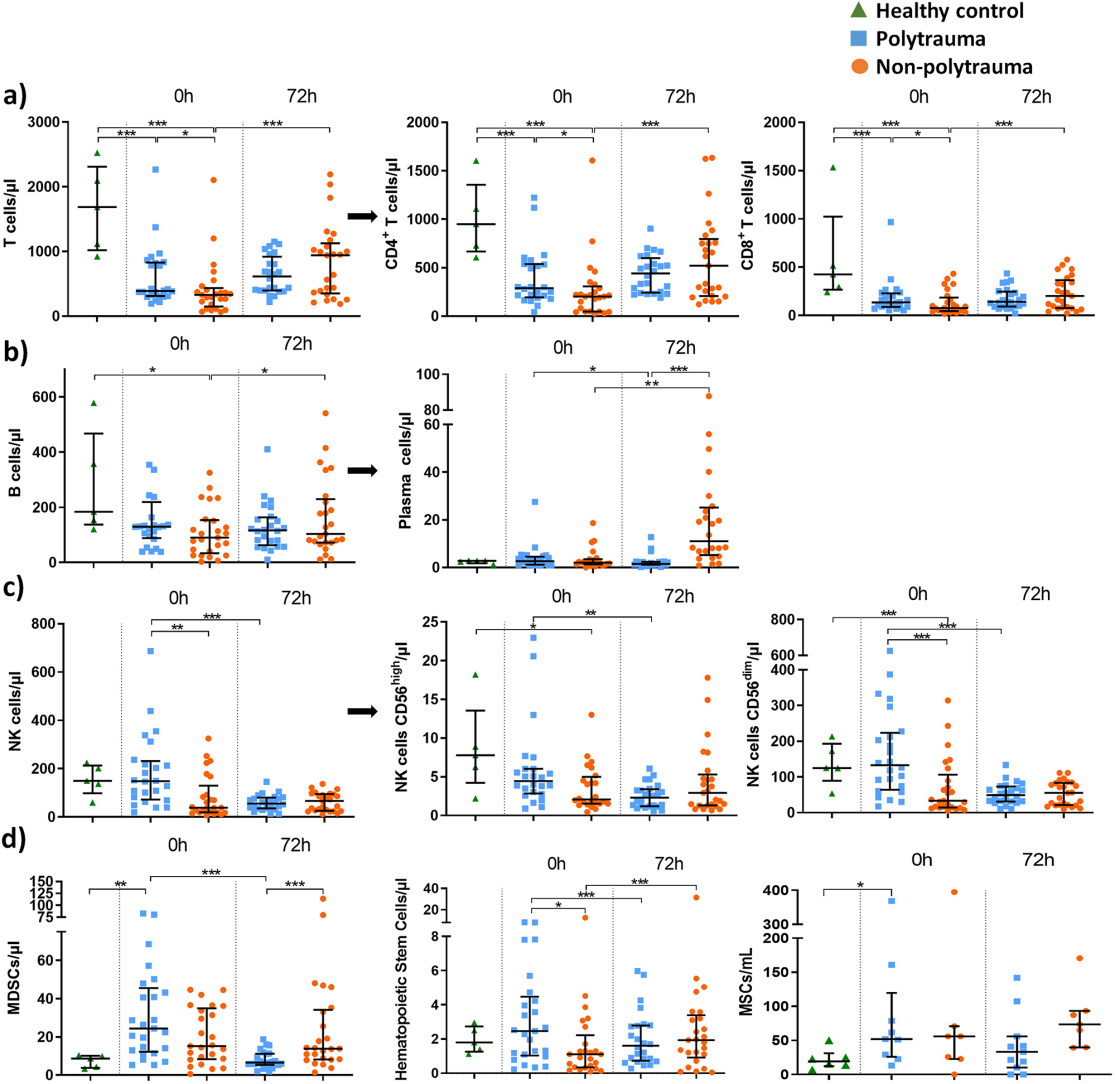
Kinetics of main lymphocyte populations and others. (**a**) T cells and CD4/CD8 subsets, (**b**) B cells and plasma cells, (**c**) NK cells and the subsets based on CD56 expression, (**d**) MDSCs (Myeloid-derived suppressor cells), hematopoietic stem cells and MSCs (mesenchymal stromal cells)

Despite the leucocytosis, lymphocyte populations decreased massively early after the SIRS onset. Total lymphocyte numbers dropped below normal levels up to 72 h in both SIRS groups (Fig. 2a). All major T cell subpopulations (i.e., CD4^pos^CD8^neg^, CD4^neg^CD8^pos^, CD4^neg^CD8^neg^TCRγδ^pos^, CD4^neg^CD8^neg^TCRγδ^neg^) decreased from SIRS onset (similarly in both groups). The ratio of CD4^pos^:CD8^pos^ remained steady (close to 2) in all groups.

Regarding the number of B cells, it decreased slightly during SIRS (Fig. 2b). Meanwhile, plasma cell numbers increased after 72 h, but only in NP SIRS patients (Fig. 2b).

Total NK cells population and its subsets (according to the expression of CD56) decreased after SIRS onset in the NP (Fig. 2c). In contrast, PT presented similar values of total NK cells at 0 h compared to HD but decreased after 72 h achieving similar values to the NP group.

### Kinetics of other populations

Focusing on less frequent circulating populations, like MDSCs, they were increased in PT early after SIRS (Fig. 2d). However, in NP, this population remained high at 72 h.

Cells commonly identified in bone marrow, such as hematopoietic stem cells (HSCs) and MSCs were identified in PB (Fig. 2d). HSC levels changed during SIRS in each disease group, with an early increase observed in the PT group compared to the NP group. MSCs increased in SIRS patients during the first hours in the PT group, whereas in the NP group increased as the SIRS advanced.

One of the crucial factors in leukocyte-endothelial transmigration and cell mobilization and recruitment is CD62L. During SIRS, there was an increase in the expression of this membrane protein in most of the studied populations (Figure S1b, Additional File 1).

### Cytokine analysis

**Fig. 3:**
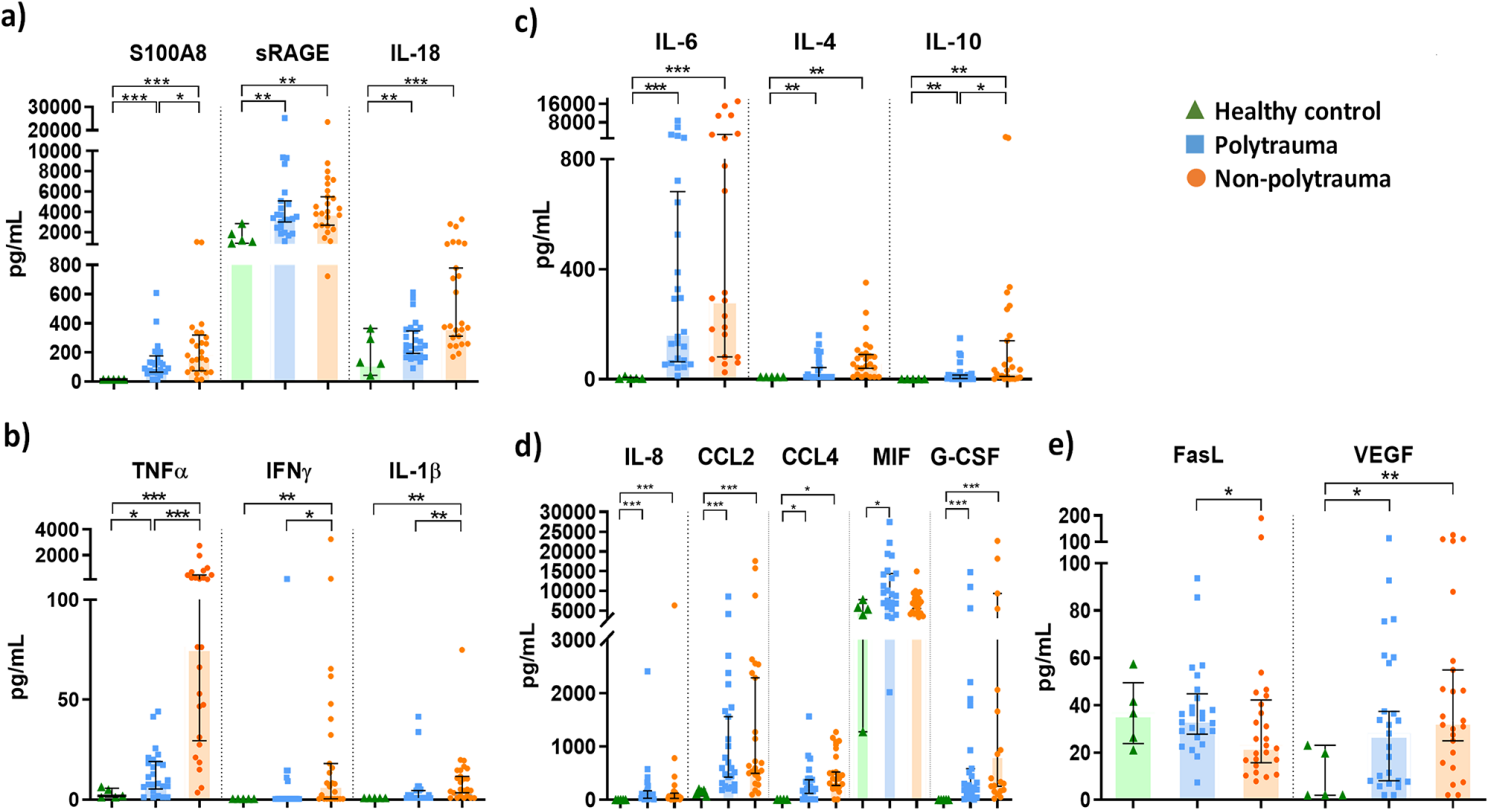
Concentration of cytokines, chemoattractant proteins and other factors in plasma from SIRS patients at the ICU admission. (**a**) Alarmins and acute phase proteins, (**b**) pro-inflammatory cytokines, (**c**) regulatory and anti-inflammatory cytokines, (**d**) chemokines, (**e**) other factors

After SIRS onset, there was an increase in most cytokines in both SIRS groups (Fig. 3). In general terms, NP patients showed higher concentrations than the PT group. Both SIRS groups showed an increase in alarmins and proinflammatory cytokines such as S100A8, sRAGE, TNFα and IL-18 compared to HD (Fig. 3a, b). NP presented higher values of both pro- (TNFα, IFNγ, IL-1β) and anti-inflammatory cytokines (IL-4, IL-10) (Fig. 3b, c). The concentration of chemokines in both disease groups increased (IL-8, CCL2, CCL4 and G-CSF) (Fig. 3d). Interestingly, MIF only appeared elevated in PT comparing to HD (p-value = 0.02). Other cytokines (IL-2, IL-12, CCL3/MIP-1a and CXCL1/GROα) were only detected in a few patients at SIRS onset. The VEGF concentration increased (Fig. 3e), whereas the apoptotic factor FASL decreased during SIRS (Figure S2, Additional File 1). The concentration of these cytokines returned close to normal values in most patients after 72 h (Figure S2, Additional File 1).

### Characterization and quantification of EVs

**Fig. 4:**
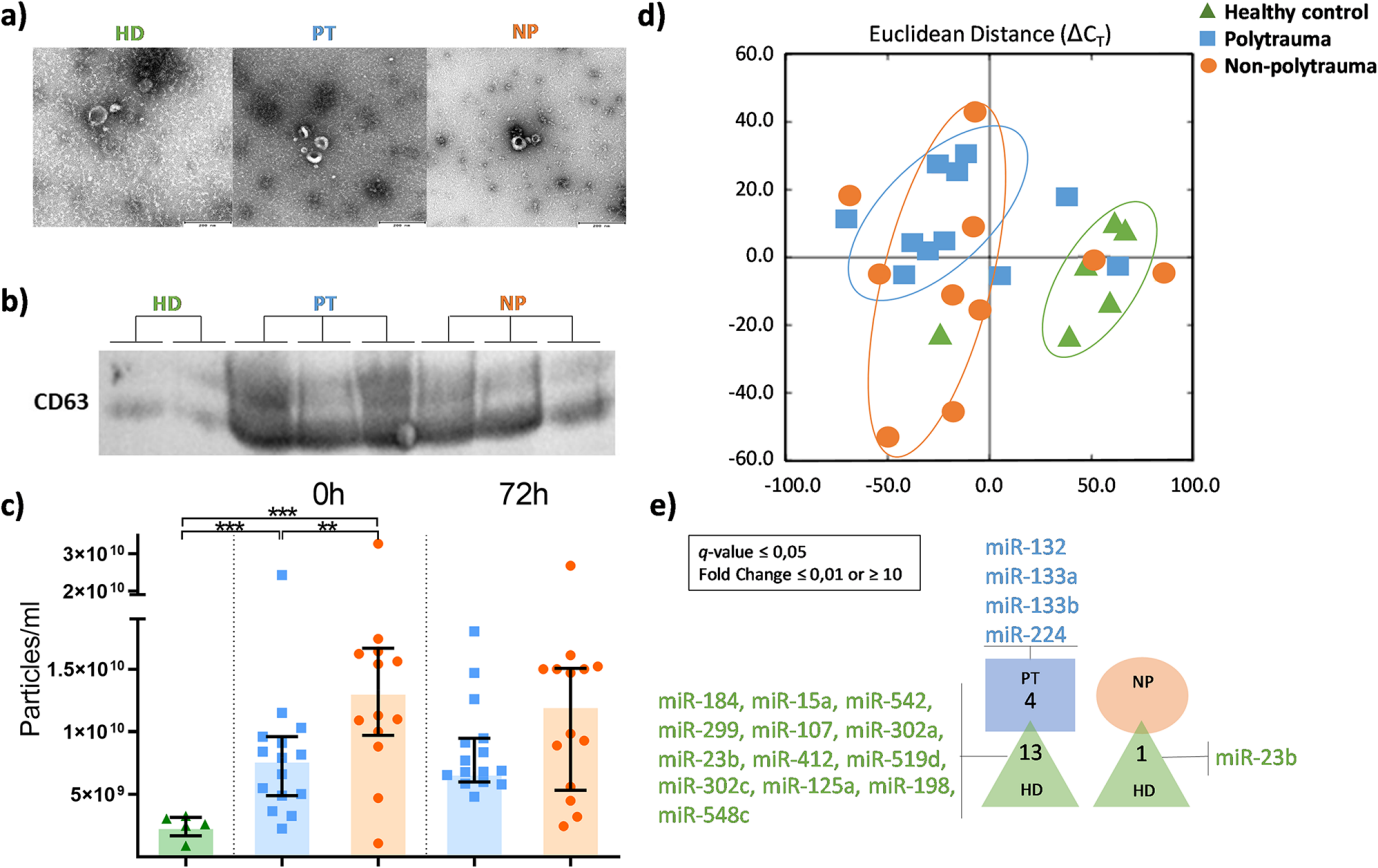
Characterization and quantification of plasma extracellular vesicles (EVs) and study of their microRNA content. (**a**) All EVs presented a classical round shape with a concave central depression. (**b**) WB results showed the CD63 expression in the EVs. (**c**) Concentration of EVs in plasma was determined using NTA technology. (**d**) Unsupervised Principal Component Analysis representation of samples according to the microRNA content. (**e**) List of microRNAs that accomplish the statistical criteria of q-value: ≤ 0,05 and present a Fold Change ≤ 0,01 or ≥ 10 in the pair-wise analysis

All patient-derived EVs displayed their characteristic spherical shape with a concave central depression on TEM (Fig. 4a) as well as the expression of CD63 by WB (Fig. 4b). The NTA analysis of plasma EVs showed similar mean sizes in both SIRS groups and HD, around 190 nm. Specifically, median and interquartile range (IQR) were 188 nm (175–208), 193 (172–214) and 185 nm (163–207), for PT, NP patients and HD, respectively. These values remained similar after 72 h. Regarding EV plasma concentration, SIRS patients presented a higher concentration of EVs compared to plasma from HD. NP patients showed higher plasma concentration compared to PT patients (Fig. 4c).

### Analysis of EV-microRNA content

Samples were clustered depending on their miRNAs expression pattern (Fig. 4d). In the Principal Component Analysis of all 370 studied miRNAs, HD samples clustered separately from most SIRS samples. Comparing both SIRS groups, EVs from PT presented a more homogeneous miRNAs content than the NP group (Fig. 4d).

Given the substantial number of differentially expressed miRNAs (125), we refined our study to those exhibiting a FC of ≤ 0.01 or ≥ 10. This adjustment resulted in 17 miRNAs differentially expressed, where 4 (mir-132, miR-133a, miR-133b, and miR-223) of them were overexpressed in the PT group (Fig. 4e). Conversely, EVs from NP showed no significant differences compared with PT and compared to HD only miR-23b was downregulated in the NP group. The main target genes of the differentially expressed miRNAs in PT patients in the miRPath and miRBase databases are summarized in Table S4 (Additional File 1).

### Correlation of biological findings with outcomes

**Fig. 5:**
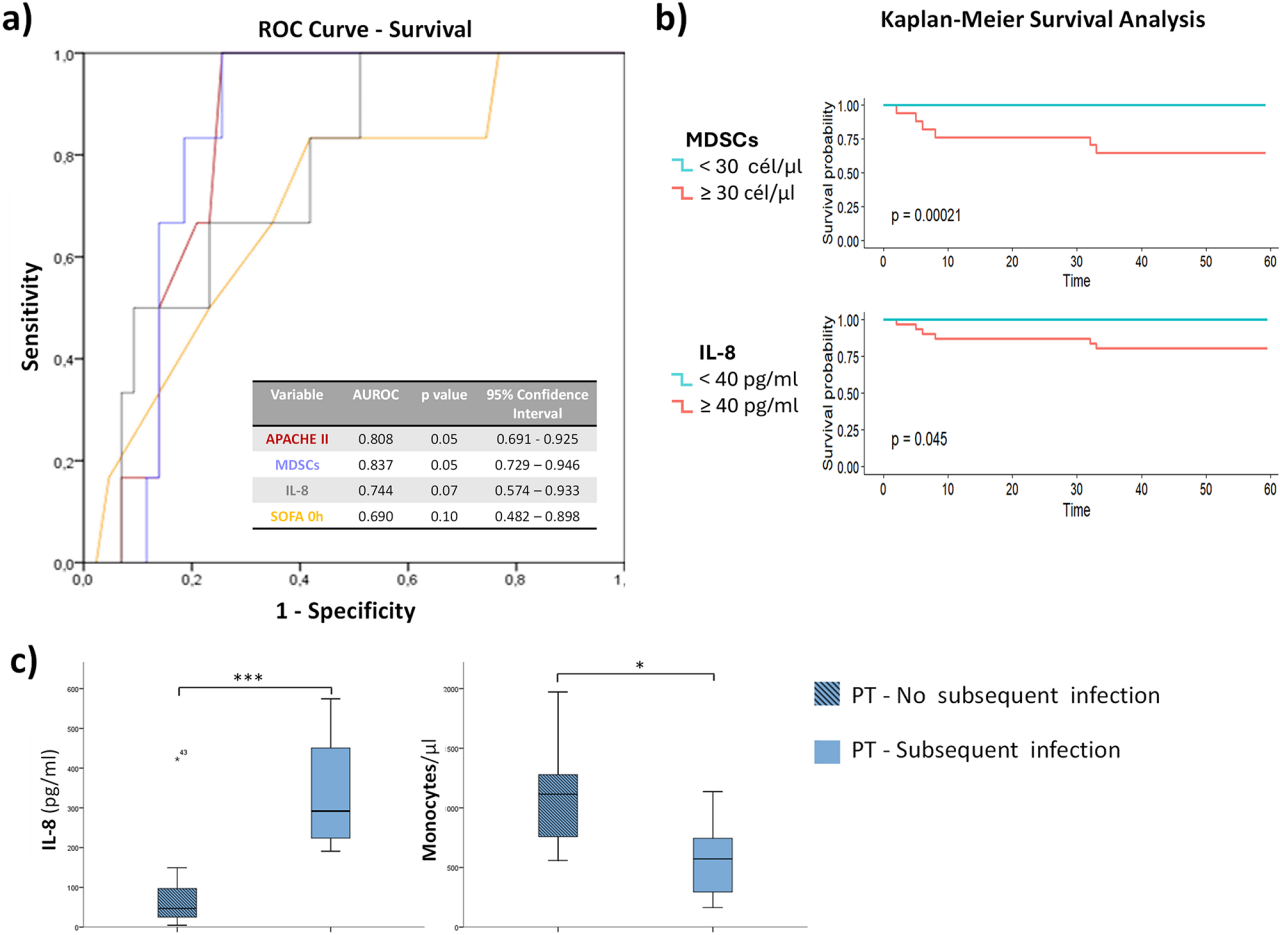
Prognostic value of potential variables for survival and subsequent infection determination. Values of these variables achieve similar discrimination power to that of the APACHE II score system in all SIRS patients, following different statistical approaches: (**a**) ROC curve and (**b**) Kaplan-Meier survival analysis. (**c**) After polytrauma, high concentrations of IL-8 and low monocyte counts are related to higher infection susceptibility

ROC curve analysis indicated that MDSCs counts and IL-8 concentrations were associated with overall survival in all SIRS patients (Fig. 5a). Area under curve (AUC) values of these markers were 0.837 and 0.744, respectively, achieving similar values to the APACHE II (Acute physiology and chronic health evaluation II) score (0.808). Kaplan-Meier analysis corroborated ROC curve results in terms of measuring the discrimination capacity of these markers (Fig. 5b). No strong direct correlations were observed among the remaining study variables (cytokines, EV levels, miRNA content) and standard clinical parameters (all those compiled in Table S1), including analysis across different time points. This lack of associations likely reflects the multifactorial and redundant nature of the inflammatory response. However, it was noted that in PT patients, higher release of IL-6 subsequent infections (p-value = 0.001; r^2^: 0.64) (Figure S3, Additional File 1).

No potential relationships were found after performing correlation analysis. Due to the high heterogeneity of SIRS origins between the two studied groups, a subgroup analysis was performed. In PT patients a high concentration of IL-8 and a low count of monocytes early after trauma was related to a higher predisposition to subsequent infection (Fig. 5c).

## Discussion

The primary novelty of this study is that it integrates a dynamic and multiparametric assessment of circulating cells (including leukocytes, HSCs and MSCs), cytokines and EVs in a series of patients with SIRS from 2 different origins (PT vs. NP), contributing to increase the knowledge in this complex field, and suggests some potential markers of outcomes.

SIRS is characterized by an inflammatory response primarily mediated by the leukocyte populations. Although both groups exhibit a broadly similar inflammatory profile (higher in NP), specific differences are driven by the type of insult, reflecting DAMP versus PAMP mediated pathways (and the interaction with purinergic receptors such as TLRs or NLRs – Toll or NOD like receptors and their intracellular signalling [19]), their inherent nature (e.g. affected organs), and their temporal dynamics (e.g. early intervention).” As it well known, SIRS patients exhibit an increase in total leukocytes, predominantly by the massive mobilization of neutrophil populations. In our study, this increase was higher in NP patients (up to 90% of total leukocytes). Massive extravasation and overactivation cause their degranulation and the release of cytotoxic compounds and NETs (neutrophil extracellular traps) compromising the tissue and endothelial integrity (vascular leakage) [20]. This neutrophil mobilization can be mediated by chemokines such as IL-8, CCL4 and G-CSF in the plasma of both SIRS groups. We showed that higher concentrations of IL-8 were related to a more altered neutrophil maturation/activation profile (based on CD16/CD62L, respectively), and higher SOFA (sequential organ failure assessment) values in polytrauma patients. Previous studies have reported that this dysregulation is associated with the severity of trauma, worse outcomes, and higher susceptibility to infectious complications [21, 22].

Due to the acuteness of the SIRS response, innate immune cells assume a pivotal role in the early pathophysiology. Monocytes characterized by their ability to phagocyte, antigen presentation, elevated cytokine production, and differentiation into macrophages within the tissues play a crucial role during SIRS acting as pivotal regulators of inflammatory dynamics, with implications for either the amplification (M1 phenotype) or resolution of systemic inflammatory responses (M2 phenotype) [23–25]. In NP patients, the iMo population increased [26, 27]. iMo are considered the most proinflammatory subset owing to their high capacity for antigen presentation and greater production of reactive oxygen species (ROS) and cytokines such as TNFα and IL-6 [28]. On the other hand, PT patients increased the monocyte population derived by the increase of CD62L^pos^ cMo subsets. This increase could be determined by the high concentration of plasma MIF, among other chemokines. It has been described that in PT patients with cranioencephalic trauma, an increase in monocytes is a good prognostic marker [29]. In this sense, we showed that PT patients with a low number of circulating monocytes were related to a higher susceptibility to infections.

Neutrophil to lymphocyte ratio has been proposed as a good parameter for clinical tracing and severity determination of SOFA, length of ICU stay, and patient survival [21, 30]. In our study, this ratio is high in both SIRS groups, particularly in NP patients, due not only to the increase of neutrophils but also to an acute reduction of total lymphocytes. Regarding cytokines, our study confirms once more the role of S100A8 and sRAGE proteins in triggering the SIRS response [21, 31]. We have also observed that concomitantly to the increase of pro-inflammatory cytokines (IFNγ, TNFα), there is not only an increase in regulatory molecules like IL-6, but also of those with well-known anti-inflammatory effects such as IL-4 and IL-10. This anti-inflammatory reaction, referred to as CARS (Compensatory Anti-inflammatory Response Syndrome), is perceived as a mechanism for controlling the exacerbated inflammation and for favoring tissue homeostasis recovery [32, 33]. The increase of inflammasome-derived cytokines such as IL-1β and IL-18 is associated with the promotion of apoptosis under inflammatory conditions [34, 35]. Conversely, the decrease of FASL in those patients with SIRS from infectious origin may be related to the promotion of cell surveillance after the disease onset [36]. These two opposed signals show the dynamics and balance in cell death signalling, which could be crucial to control tissue viability and MODS (multiple organ dysfunction syndrome) evolution.

Migration is conditioned by the massive secretion of cellular factors. Interestingly, most chemokines remained at similar levels between PT and NP patients, except for CCL4 and MIF. However, circulating leukocyte counts did vary between both groups. Thus, the control of leukocyte migration may also be determined by other factors. A fundamental factor in the pathophysiology of SIRS is the alteration in vascular integrity (e.g. endothelial integrity where VEGF plays a crucial role). An uncontrolled activity of the immune system alters the state of the microvasculature of organs such as the lungs [37]. The endothelium is also sensitive to hypercytokinemia causing its non-specific activation and favoring cell and plasma extravasation even to initially unaffected tissues [38]. CD62L (L-selectin), a key marker involved in the regulation of transendothelial migration and essential for leukocyte-endothelium interaction, is upregulated during SIRS in most circulating cell populations. Inflammatory conditions following trauma, characterized by a dysregulated chemokine release, trigger uncontrolled leukocyte migration that can affect even non-injured tissues. Therefore, regulating both immune cell migration and overactivation within the microenvironment is crucial to minimizing the collateral effects associated with SIRS [39]. Another critical situation occurs when circulating populations do not recover within a few days after the onset of SIRS. This lack of renewal from the bone marrow can induce a state of immunodeficiency [40]. During this emergency myelopoiesis, the hematopoietic niche is altered, and HSCs can enter a state of exhaustion [40, 41]. Regarding this, the HSCs population increased in circulation in PT compared to NP patients. This release can be induced not only by G-CSF signaling, but also by the fracture of large bones and the release of bone marrow cells into the bloodstream [42, 43]. Similarly, the population of MSCs increased its concentration in circulation at the onset of SIRS in the PT group as previously reported [44]. This mobilization of MSCs can be interpreted as an inflammatory regulatory response due to their immunomodulatory capabilities shared with MDSCs [45, 46]. Both populations interact with other leukocyte populations promoting an immunosuppressive effect through the secretion of anti-inflammatory cytokines (TGFβ and IDO) [47, 48]. Overall, MDSCs counts along with the IL-8 concentration are good markers for determining the prognostic of SIRS patients in terms of survival. Additionally, while IL-8 is already recognized for its clinical relevance, MDSCs may provide additional insights into immune dysregulation states Moreover, the rapid mobilization of dysfunctional neutrophils (induced by the IL-8 rise) as well as monocytes with low HLA-DR expression may underlie a combined innate-adaptative immunoparalysis that can increased infection susceptibility in PT patients [49].

Regarding the role of circulating plasma EVs, we have observed a significant increase by 2.5-fold after a PT and up to 5-fold in patients under infection processes. Similar results have been described in patients with burns or severe pneumonia [50, 51]. Interestingly, EV concentrations remained high after 3 days since the SIRS onset, as it has been observed after trauma [52]. Referring to their miRNA content, we obtained preliminary findings indicating a potential influence of SIRS etiology on miRNA profiles, as some samples appeared to cluster by their origin. We have identified some miRNAs (miR-132, miR-133a, miR-133b, miR-224) that are overexpressed in EVs from PT patients. These miRNAs target genes involved in cell migration and immune synapses regulation that could interfere with leukocyte recruitment, tissue infiltration and immune activation (Figure S4). An increase in miR-133a, observed in the polytrauma group, has been associated with poor prognosis in critically ill patients [53]. Upregulation of miR-132 in keratinocytes after injury may be critical for facilitating tissue recovery, which highlights its potential value as a therapeutic candidate to modulate inflammation and promote repair [54]. Concurrently, upregulation of miR-132 in keratinocytes may be critical for facilitating the tissue recovery after injury [55]. Among the targets of these miRNAs are ATM, RB1M2, KRAS or SP1 as key modulators of cell cycle, proliferation, differentiation, and inflammatory processes. These observations open a path for further investigation to confirm their biological and clinical relevance. The balance among all factors involved in SIRS, such as cytokines and extracellular vesicles, is crucial in determining leukocyte responses. These components both conditionate and are influenced by the surrounding microenvironment. The microenvironment plays a key role in shaping inflammatory responses, and studying chronic inflammation can provide valuable insights to better understand immune regulation in acute conditions such as SIRS [56].

Our study has several limitations, including high heterogeneity in SIRS triggers, particularly in the NP group, and other intrinsic sample factors such as time sample collection and age-related conditions. This could also contribute to increased variability in the results, with some extreme values deviating from the overall mean. Nevertheless, thanks to the differences observed within the septic patient group, our work may serve as a starting point for future and more specific studies on subgroups stratified by infection origin. The number of patients enrolled is reduced due to the hard accessibility to samples of critically ill patients, and this may have limited some of the findings and potential correlations.

## Conclusions

In summary, our study shows the dynamic kinetics of multiple cell subsets, cytokines and EVs during SIRS, showing differences between SIRS initiating primary event (PT versus NP patients) and some correlations between biological findings and outcomes that require further validation studies.

## Electronic Supplementary Material

Below is the link to the electronic supplementary material.

Supplementary Material 1

## Data Availability

The datasets supporting the results of this article are available from the corresponding author upon reasonable request. Be aware that there are ethical and legal restrictions on sharing the original study datasets. The electronic health records data cannot be shared publicly because it consists of personal information from which it is difficult to guarantee the identification (Law 03/2018 from Spanish Government - BOE-A-2018-16673).

## Abbreviations

ALT: Alanine transaminase
APACHE: Acute physiology and chronic health evaluation
APC: Antigen-presenting cells
AST: Aspartate transaminase
AUC: Area under curve
CARS: CCLn: C-C motif chemokine ligand n
CD: Cluster of differentiation
cMo: Classical monocytes
CRP: C-reactive protein
CRRT: Continuous renal replacement therapy
DAMPs: Damage-associated molecular patterns
DCs: Dendritic cells
EDTA: Ethylenediaminetetraacetic acid
EVs: Extracellular vesicles
G-CSF: Granulocytic colony stimulating factor
GGT: Gamma-glutamyl transferase
HD: Healthy donors
HSCs: Hematopoietic stem cells
ICU: Intensive care unit
IQR: Interquartile range
ISS: Injury severity score
IL-n: Interleukin
iMo: Intermediate monocytes
LDH: Lactate dehydrogenase
LOS: Length of stay
mDCs: Myeloid dendritic cells
MDSCs: Myeloid-derived suppressor cells
MFI: Median fluorescence intensity
MIF: Macrophage migration inhibitor factor
miRNAs: microRNAs
Mo: Monocytes
MODS: Multiple organ dysfunction syndrome
MSCs: Mesenchymal stromal/stem cells
ncMo: Non-classical monocytes
NETs: Neutrophil extracellular traps
NK: Natural killer
NLRs: NOD-like receptors
NP: Non-polytrauma group
NTA: Nanoparticle-tracking analysis
PAMPs: Pathogen-associated molecular pattern
PBMCs: Peripheral mononuclear cells
PCT: Procalcitonin
pDCs: Plasmacytoid cells
PT: Polytrauma group
ROC: Receiver Operating Characteristic
SIRS: Systemic inflammatory response syndrome
SOFA: Sequential organ failure assessment
TEM: Transmission electron microscopy
TLRs: Toll-like receptors
TNFα: Tumour necrosis factor alpha
TP INR: Prothrombin time international normalized ratio
WB: Western blot
