# Supplementary material for: Immune cell dynamics, cytokines, and extracellular vesicles in systemic inflammatory response syndrome (SIRS): a multiparametric analysis
Source: J Transl Med. 2025 Nov 21;23:1331. doi: 10.1186/s12967-025-07327-z (PMC12639914; doi:10.1186/s12967-025-07327-z)
Supplement: Supplementary file 1 — Supplementary Material 1 [file 12967_2025_7327_MOESM1_ESM.pdf]

Supplementary material

Table S1. Demographic and clinical data of the SIRS patients at the study admission and healthy donors.

	**Healthy (n=21)**	**Polytrauma (n=25)**	**Non-Polytrauma (n=25)**	p-value
		Demographics and clinical outcome		
Age	56 (53-60)	50 (44-62)	66 (53-73)	0.01
Male/Female	12/9	23/2	15/10	0.009
APACHE II	-	18 (14-24)	22 (17-26)	ns
ISS	-	26 (22-29)	-	-
SOFA at admission	-	7 (6-10)	8 (6-10)	ns
ICU stay	-	16 (11-21)	5 (4-10)	≤ 0.001
Total hospital stay (days)	-	27 (18-37)	11 (7-25)	≤ 0.001
Death (at 28th day)	-	2/25 (8)	4/25 (16)	ns
		Therapy and organ replacement		
Mechanical ventilation (%)	-	24 (96)	22 (88)	ns
Fluid resuscitation (%)	-	25 (100)	23 (92)	ns
Vasopressors (%)	-	23 (92)	23 (92)	ns
CRRP (%)	-	1 (4)	5 (20)	ns
Platelet transfusions (%)	-	5 (20)	5 (20)	ns
Erythrocyte transfusion (%)	-	14 (756)	5 (20)	0.019
		Leukocytes and inflammatory markers		
	Reference range			
Leukocytes (x10^3/µl)	4.5-10.8	16 (12-18.1)	20.3 (12-28.6)	0.013
Neutrophils (x10^3/µl)	1.4-6.5	13.1 (9.3-15.2)	14.9 (9.6-21.8)	0.007
Lymphocytes (x10^3/µl)	1.2-3.5	1 (0.8-1.6)	0.6 (0.5-1)	ns
Monocytes (x10^3/µl)	0.3-0.9	1 (0.6-1.2)	0.7 (0.5-1)	ns
CRP (mg/dL)	0-0.5	1.5 (0.4-5.2)	24.9 (18.8-30)	≤ 0.001
PCT (ng/mL)	0-0.5	2.1 (1.3-13)	32.3 (15.3-83)	≤ 0.001
		Markers of organ damage and function		
Lactate	≤ 2 mmol/L	2.8 (2-3.9)	2.1 (1.6-3)	0.045
AST	0-40 U/L	221 (76-505)	131.5 (66.5-646.5)	ns
ALT	0-41 U/L	73 (35-233)	22 (16-44.5)	0.004
GGT	0-60 U/L	38 (17-51.5)	75 (25-101.5)	ns
Bilirubin (mg/dL)	0.15-1.2	0.5 (0.4-0.7)	0.8 (0.4-1.1)	0.028
Creatinine (mg/dL)	0.7-1.2	1 (0.8-1.2)	1.6 (1.1-2.8)	0.002
Urea (mg/dL)	17-49	35 (31-41)	64 (47-104)	≤ 0.001
		Coagulation markers		
Platelets (x10^3/µl)	150-450	174 (148-226)	188 (137.5-249.3)	ns
Fibrinogen (mg/dL)	200-400	209 (172.6-242.8)	533 (401-702.6)	≤ 0.001
TP INR (s)	0.8-1.2	1.2 (1.1-1.3)	1.3 (1.2-1.5)	ns
		Other markers		
LDH (U/L)	135-280	491 (305-708)	208 (168-257)	≤ 0.001
Glucose (mg/dL)	76-110	181 (149-208)	148 (105-180)	0.008

Note: The values represented correspond to the median and interquartile range, or the number of subjects followed by the percentage in parentheses on the first day of inclusion in the study. Treatment monitoring was performed for the first 3 days. APACHE II: Acute Physiology and Chronic Health disease Classification System II, ISS: Injury Severity Score, SOFA: Sequential Organ Failure Assessment, CRRT: Continuous Renal Replacement Therapy, CRP: C-Reactive Protein, PCT: Procalcitonin, AST: Aspartate Transaminase, ALT: Alanine Transaminase, GGT: Gamma-glutamyl transferase, TP INR: Prothrombin Time International Normalized Ratio, LDH: Lactate Dehydrogenase.

Table S2. Conjugated antibody combinations and flow cytometry strategy used for identifying circulant populations.

a)

Tube	FITC	PE	PE-Cy7	PE CF594	APC	APC-R700	APC-H7	PerCP-Cy5	BV421	BV510	BV605	BV650	BV711	BV786
**MoDC**	**CD1c**	**Slan/ FcεRI**	**CD33**	**CD34**	**CD303/ CD300e**	**CD45**	**CD14**	**CD36**	**CD141**	**CD5**	**CD192**	**CD62L**	**HLA-DR**	**CD16**
**LST**	**CD8/ λ Chain**	**CD56/ κ Chain**	**TCRγδ/ CD19**	-	**CD3**	-	**CD38**	**CD5**	**CD4/CD20**	**CD45**	-	-	-	-
**MSCs**	**CD34**	**MSCA1**	**CD146**	-	**CD271**	**Viability Stain 700**	**CD73**	**CD45**	**CD105**	**HLA-DR**	**CD10**	-	**CD13**	**CD90**

b)

Populations	Membrane Markers							
**Neutrophils**	**SSCA high**	**FSCA high**	**CD45+**	**HLADR-**	**CD14-**			
Mature						CD16+		
Imm. CD16-/CD62L+						CD16 dim/-	CD62L+	CD33+
Imm. CD16-/CD62L-						CD16-	CD62L-	CD33+
**Eosinophils**	**SSCA high**	**FSCA high**	**CD45+**	**CD16-**				
**Basophils**	**SSCA low**	**FSCA low**	**CD45 dim**	**FcERI+**	**CD62L +**			
**Monocytes (Mo)**	**SSCA dim**	**FSCA dim**	**CD45+**	**HLADR+**	**CD33+**	**CD14+**		
**Classical Mo (cMo)**						**CD14+**	**CD16-**	**CD192+**
cMo FcERI+/CD62L-							FcERI+	CD62L-
cMo FcERI+/CD62L+							FcERI+	CD62L+
cMo FcERI-/CD62L-							FcERI-	CD62L-
cMo FcERI-/CD62L+							FcERI-	CD62L+
**Intermediate Mo (iMo)**	**SSCA dim**	**FSCA dim**	**CD45+**	**HLADR+**	**CD33+**	**CD14+**	**CD16+**	
**Non-classical Mo (ncMo)**	**SSCA dim**	**FSCA dim**	**CD45+**	**HLADR+**	**CD33+**	**CD14 dim/-**	**CD16+**	
ncMo Slan+/CD36-							SLAN+	CD36-
ncMo Slan+/CD36+							SLAN+	CD36+
ncMo Slan-/CD36-							SLAN-	CD36-
ncMo Slan-/CD36+							SLAN-	CD36+
**Dendritic cells (DCs)**	**SSCA dim**	**FSCA dim**	**CD45+**	**HLADR+**	**CD16-**			
Myeloid DCs (mDCs)						CD1c dim/+	CD33+	CD141+
mDCs CD14dim						CD14 dim	CD5-	
mDCs CD5-/CD14-						CD14-	CD5-	
mDCs CD5+						CD14-	CD5+	
mDCs CD141+						CD141+		
Plasmacytoid DCs (pDCs)						CD303+	CD33-	CD1-
DCs Axl+						CD303+	CD33+	CD1-
**MDSCs**	**SSCA dim**	**FSCA dim**	**CD45+**	**HLADR dim/-**	**CD33+**	**CD14+**	**CD16-**	**CD192-**
**Hematopoitetic stem cells**	**SSCA low**	**FSCA low**	**CD45 dim**	**HLADR+**	**CD34+**			
**T cells**	**SSCA low**	**FSCA low**	**CD45+**	**CD3+**				
CD4					CD4+	CD8-	TCRgd-	
CD8					CD4-	CD8+	TCRgd-	
TCRgd+					CD4-	CD8-	TCRgd+	
TCRgd-					CD4-	CD8-	TCRgd-	
**B Cells**	**SSCA low**	**FSCA low**	**CD45+**	**CD3-**	**CD19+**	**HLA-DR+**		
Lambda							Igλ+	
Kappa							Igk+	
Cél. Plasmática					CD20-	CD38+		
**NK Cells**	**SSCA low**	**FSCA low**	**CD45+**	**CD3-**	**CD19-**	**CD16+**		
CD56high							CD56+	
CD56dim							CD56 dim	
CD56low							CD56 dim/-	

Table S3. Additional information of protocols followed for plasma extracellular vesicle isolation, characterization and the analysis of EVs-microRNA.

**Plasma EVs isolation**	After centrifuging the PB tubes at 800 g for 10 min and the plasma at 3,000 g for 20 min twice to remove remanent cell debris, plasma was aliquoted in 0.5 mL cryovials and kept at -80ºC in cryovials for further steps. Then, after thawing the cryovials, plasma was centrifuged at 3,800 g for 30 min. The supernatant phase was filtered using a 0.22 µm membrane for the exclusion of larger molecules. Before ultracentrifugation, the starting volume of plasma was measured and considered for downstream calculations. Plasma was diluted with double-filtered PBS (Phosphate-Buffered Saline, Gibco ref. 14190-094) and ultracentrifuged at 100,000 g for 120 min at 4ºC in the Optima L-90K ultracentrifuge (Beckman Coulter) with the 70Ti rotor (Beckman Coulter) and the CP100NX (Himac) with the P70AT-1648 rotor. Then, supernatants were removed, and a washing step was performed by ultracentrifuging again with PBS at 100,000 g for 70 min at 4ºC. Finally, the precipitated was resuspended in specific diluents depending on the type of analysis (specified below).
**EVs Morphology study**	For the morphological study the isolated EVs were resuspended in 50 µL of PBS and stored at -80°C until analysis. The TEM study was performed at the Microscopy Service of the Universidad Autónoma de Madrid using a FEITecnai G2 electron microscope (Spirit Biotwin). For image capture, the samples were fixed with 2% paraformaldehyde and 1% glutaraldehyde on carbon-coated grids (Formvar). A uranyl-oxalate solution was used as a contrast solution before imaging with a digital camera.
**EVs quantitation and size determination**	EVs were isolated from 1 ml of plasma and finally resuspended in a total of 150 µl of PBS. Their quantification and median diameter determination was performed by the NTA using a Nanosight NS300 instrument equipped with an sCMOS camera, 488 blue laser and, NTA analysis software v.3.4 was used. All captures were performed in the continuous flow chamber under the conditions of 23-25ºC temperature at 25 FPS (frames per second). This analysis was performed in the SOFT ICMAB/U6-Nanbiosis service of the Institute of Materials Science of Barcelona (ICMAB-CSIC).
**Western Blot study**	After ultracentrifugation, EVs were resuspended in 150 µl of Lysis Buffer (RIPA, ChemCruz ref. sc-24948) with protease inhibitors (200mM PMFS, 100 mM sodium orthovanadate). After 20 min on ice and vortexing, samples were centrifuged at 13,000 rpm for 15 min and the supernatant was collected and stored at -20ºC. Denatured proteins (at 100ºC for 10 min) were loaded in an SDS-PAGE gel (12% acrylamide). Wet transference was performed by the sandwich technique onto a PVDF membrane. After transference, the membrane was removed and blocked with non-fat milk (Blotting-Grade Blocker, Bio-Rad ref. 170-6404) for 1 hour. Then, the membrane was incubated overnight with a primary anti-CD63 antibody (rabbit anti-human, System Biosciences ref. EXOAB-CD63A-1). Following this, a standard secondary antibody (anti-rabbit conjugated with horseradish peroxidase) was added for 2 hours, and the membrane was revealed in the iBright Imaging Systems (Invitrogen).
**EVs-microRNA content analysis**	Under a conventional Trizol-Chloroform method, a reverse transcription (RT) reaction was performed following the indications of the Megaplex RT Reactions kit (Applied Biosystems ref. 4366596). Subsequently, a preamplification step was required using the Megaplex PreAmp reagents (Applied Biosystems ref. 4399233). Finally, a quantitative PCR (RT-qPCR) reaction was performed for the quantification of multiple miRNAs using the TaqMan type A array card (Applied Biosystems) and kit reagents (TaqMan Universal PCR Master Mix, Applied Biosystems ref. 4324018).<br><br>To determine the relative expression of each miRNA per sample the analysis software DataAssist v.2.2 (Thermo Fisher) was used. The expression of each miRNA was normalized with the CT values of U6 snRNA and RNU48 (endogenous or reference controls). For the analysis of miRNA expression, the Limma package of the R software was used. In addition, values of ΔCT (ΔCT miRNA SIRS group – ΔCT miRNA Healthy Control group) and Fold Change (FC; 2-ΔCT) were calculated. Differentially expressed miRNAs between groups were those that presented a q-value (adjusted p-value) ≤ 0.05 and had a range of differential expression or Fold Change (FC) of [≤ 0.1 or ≥ 10] compared to the reference group. The miRPath v.3 (Diana Tools) and miRBase databases were used to evaluate the role of the differentially expressed miRNAs. R studio was used for KEGG and GO terms analysis and data representation based on TarBase v8.0 database.

Table S4. Genes regulated by miRNAs overexpressed by the polytrauma group respected to the control group.

GENES	CELL ACTIVITY OR PATHWAY
GSK3B, SMC1A, YWHAH, CCNB1, SMAD2, CCNA2, CDC25B, STAG-2, WEE1, CDK6, ATM, CCND1, E2F5, RBL1, RB1, EP300, CDKN1A, PRKDC, RAD21, ORC5, MDM2	Cell Cycle
CCNB1, SMAD2, STK4, SIRT1, KRAS, IKBKB, ATM, NLK, PIK3R3, CCND1, PIK3R1, PRKAA1, EP300, AKT3, SOD2, CDKN1A, PTEN, SGK3, MAPK1, GRB2, MDM2, BCL2L11	FoxO
GSK3B, YWHAH, YAP1, SMAD2, WNTSA, PPP1CC, ACTG1, AMOT, CDH1, CSNKID, CCND1, CTNNA1, FRMD6, PPP2CB, WNT3A, LATS2, PPP2RIB, CTGF	Hippo
SMAD2, THBS1, ACUR2B, E2F5, SMURF1, RBL1, SMAD5, SP1, EP300, PPP2CB, MAPK1, PPP2R1B	TGFβ signalling

Supplementary Figures

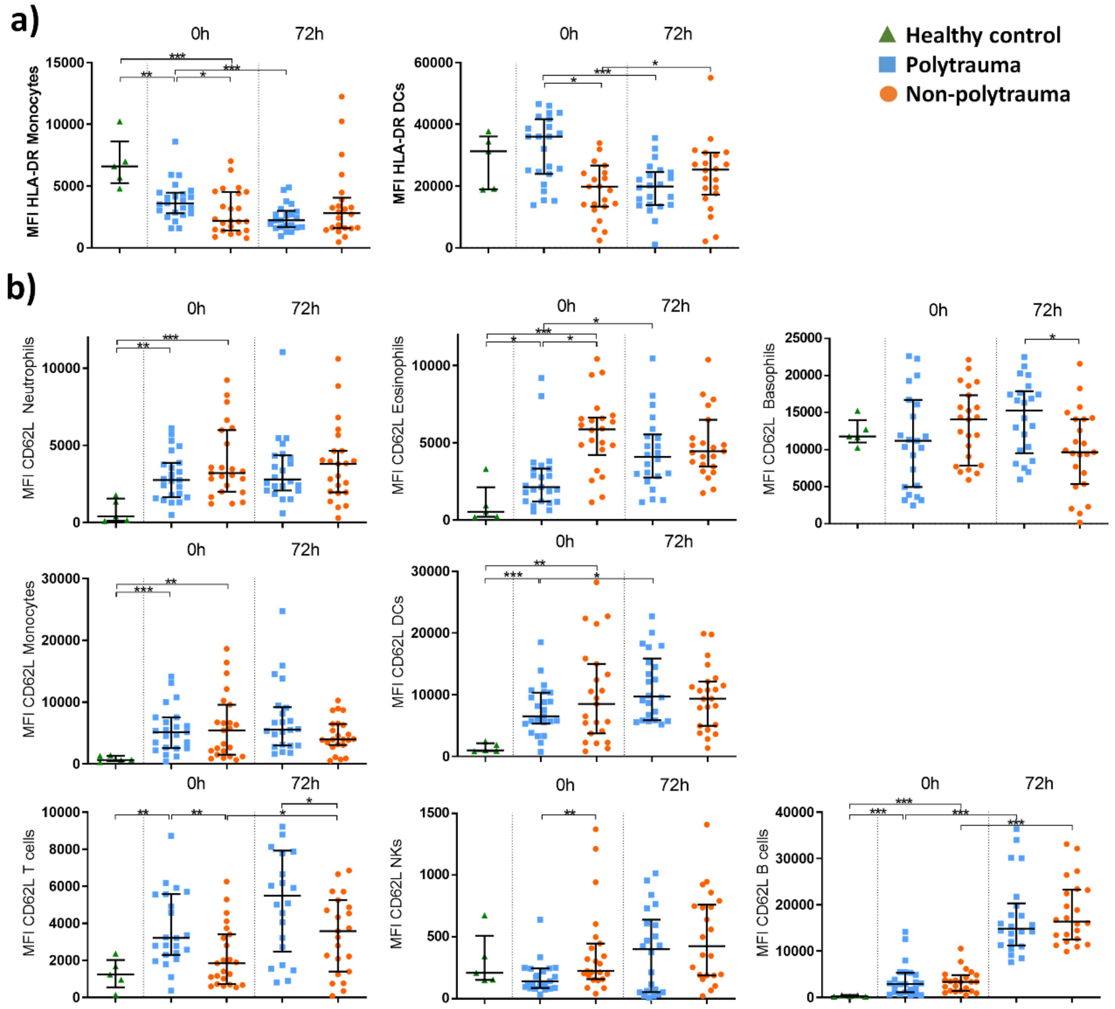

Figure S1. Expression (MFI) of a) HLA-DR in antigen-presenting cells, and b) CD62L in major leukocyte populations.

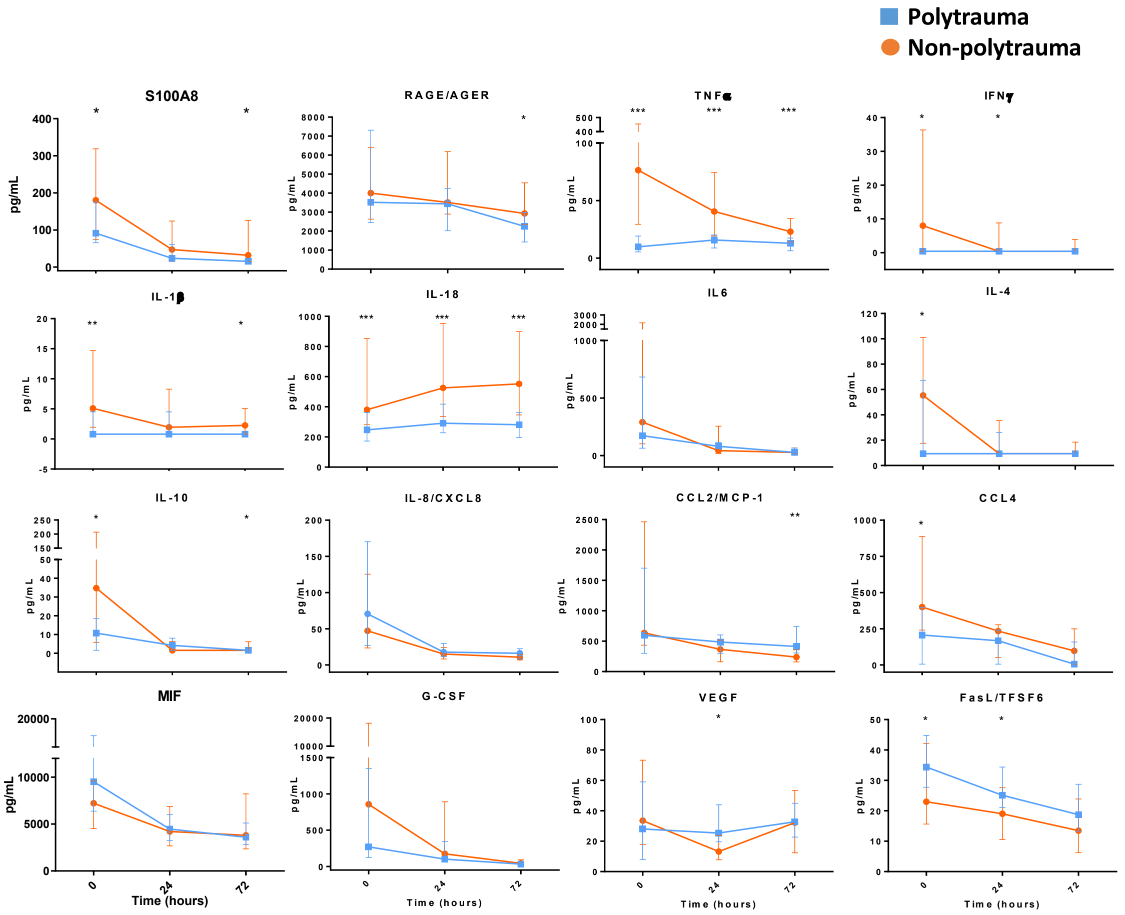

Figure S2. Concentration kinetics of cytokines, chemokines and other factors in plasma from ICU patients during the first 72 hours since the SIRS onset.

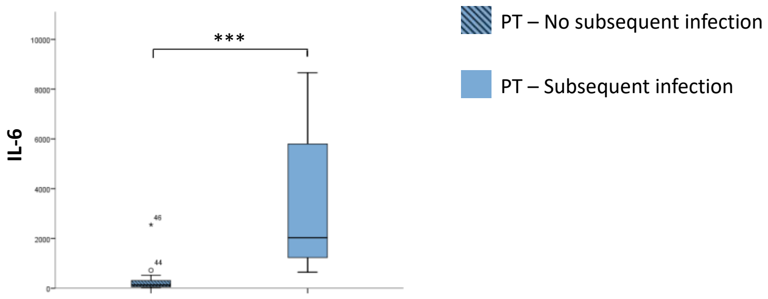

Figure S3. High concentrations of IL-6 in PT patients is related to high infection predisposition. The number of patients that presented a subsequent infection were 5.

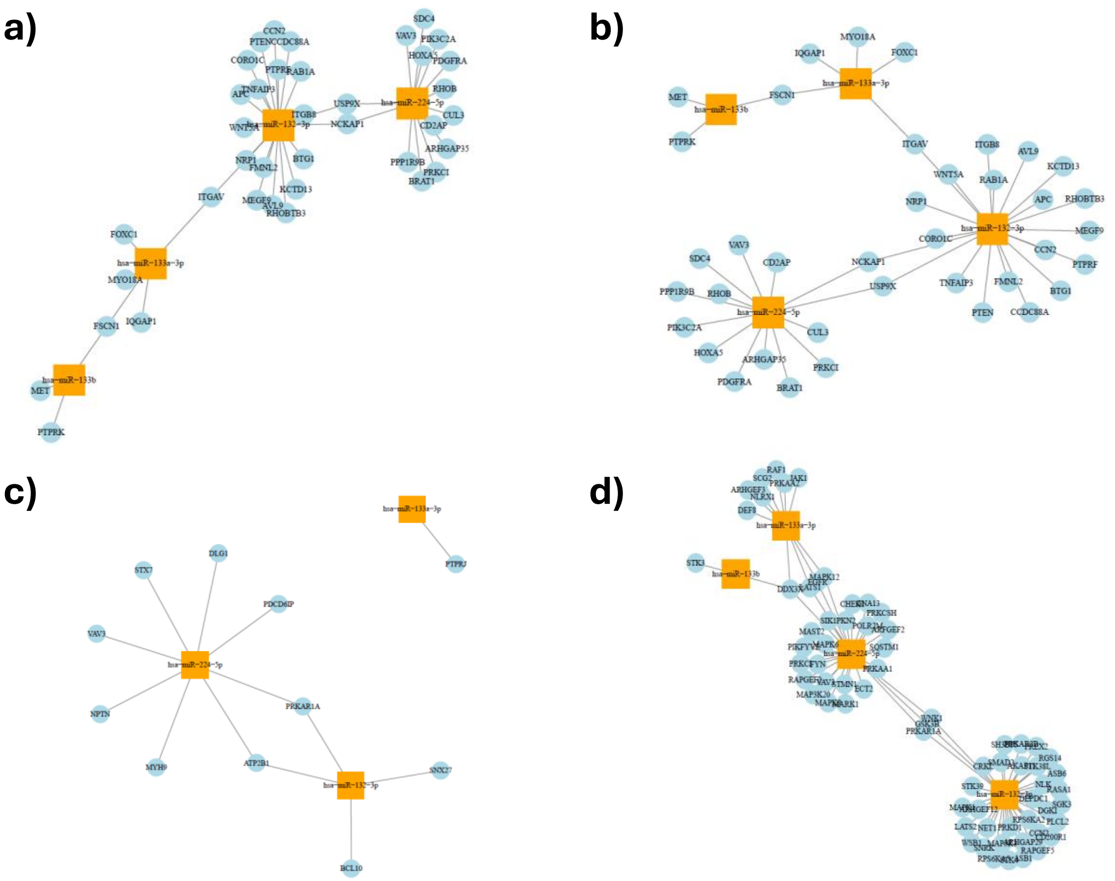

Figure S4 Network representation showing the relationship between overexpressed microRNAs (orange) of EVs from PT samples comparing to HD and their predicted target genes (blue) involved in: a) cellular migration, b) focal adhesion, c) immunological synapses and d) intracellular signal transduction based on GO terms.
